# Can equity in care be achieved for stigmatized patients? Discourses of ideological dilemmas in perioperative care

**DOI:** 10.1186/s12913-024-10580-5

**Published:** 2024-02-15

**Authors:** Maria Härgestam, Lenita Lindgren, Maritha Jacobsson

**Affiliations:** 1 Department of Nursing, Umeå University, Umeå, Sweden; 2 Department of Social Work, Uppsala University, Uppsala, Sweden

**Keywords:** Discrimination, Discourse psychology, Equity, Ideological dilemma, Perioperative care, Stigmatization, Weight bias

## Abstract

**Background:**

In the perioperative care of individuals with obesity, it is imperative to consider the presence of risk factors that may predispose them to complications. Providing optimal care in such cases proves to be a multifaceted challenge, significantly distinct from the care required for non-obese patients. However, patients with morbidities regarded as self-inflicted, such as obesity, described feelings of being judged and discriminated in healthcare. At the same time, healthcare personnel express difficulties in acting in an appropriate and non-insulting way. In this study, the aim was to analyse how registered nurse anaesthetists positioned themselves regarding obese patients in perioperative care.

**Methods:**

We used discursive psychology to analyse how registered nurse anaesthetists positioned themselves toward obese patients in perioperative care, while striving to provide equitable care. The empirical material was drawn from interviews with 15 registered nurse anaesthetists working in a hospital in northern Sweden.

**Results:**

Obese patients were described as “untypical”, and more “resource-demanding” than for the “normal” patient in perioperative care. This created conflicting feelings, and generated frustration directed toward the patients when the care demanded extra work that had not been accounted for in the schedules created by the organization and managers.

**Conclusions:**

Although the intention of these registered nurse anaesthetists was to offer all patients equitable care, the organization did not always provide the necessary resources. This contributed to the registered nurse anaesthetists either consciously or unconsciously blaming patients who deviated from the “norm”.

**Supplementary Information:**

The online version contains supplementary material available at 10.1186/s12913-024-10580-5.

## Background

Discrimination due to weight is the third leading cause of prejudice, next to age and race [1]. In contrast to more widely recognized social stigmas that have legal sanctioning to protect individuals from discrimination, there are no laws prohibiting weight discrimination. Discrimination due to weight occurs in multiple settings, including employment, healthcare facilities [2], educational institutions [3], and interpersonal relationships with friends and family [4]. Reducing health inequities is a stated goal for healthcare systems worldwide, and according to the Swedish constitution, healthcare organizations and all public organizations must work for equity [5]. Discrimination based on gender, national or ethnic origin, linguistic or religious affiliation, disability, sexual orientation, age, or other circumstances that apply to the individual is prohibited by legislation in Western societies. However, it is necessary to ask what this means in practice; and, specifically, how equity in healthcare can be achieved for a stigmatized patient group.

Globally, obesity is a growing health problem [6] which is also reflected in Health care. The increased need for medical care may challenge the future healthcare system due to various diseases associated with obesity [7]. However, this patient group describe feelings of being judged and treated differently, deviating from the standard, and being a generally accepted target of discrimination affecting the individual [1]. Similar situations involving feelings of shame and discrimination have also been described by patients with morbidities that are often regarded as self-inflicted, such as chronic obstructive pulmonary disease [8], liver cirrhosis [9], and HIV [10]. In healthcare, weight stigma and discrimination against obese patients have been well-documented by research in recent decades [11, 12]. Prejudices in society also reflect biases among healthcare professionals in primary care [13] and hospital settings [14], as well as among nursing students [15] and medical students [16], which will also affect the quality of care for obese patients [17]. On the other hand, interviews with public health nurses have described difficulties in relating to patients with severe obesity [18], and nurses in a bariatric surgery ward found it challenging to express themselves in an appropriate and non-insulting way [19]. Robstad, Soderhamn & Fegran (2020) found similar results when interviewing intensive care nurses [20].

Goffman argues that stigma can be seen as a dynamic social process attributed to certain groups or individuals based on their perceived status [21]. For example, there is a risk that overweight patients will be exposed to stereotypes and negative attitudes from healthcare personnel, such as the belief that fat people are irresponsible and ignorant about “good” health behaviors [22]. This negative attitude can affect the interaction between nurses and patients, putting the patient in an uncomfortable position, and the stigmatized individuals may internalize this discounting as self-blame and shame [22]. Scheff (2003) acknowledges shame as a social and psychological phenomenon [23], while Janoff-Bulman (1979) distinguishes between two types of self-blame: characterological versus behavioural self-blame [24]. Individuals with characterological self-blame believe they have a poor ability to control the situation and succeed. In contrast, individuals with behavioural self-blame believe that they will be able to control the outcome as long as they try harder. In the perioperative context, the primary focus lies on managing the critical physiological issues associated with a high BMI, and research on RNAs experiences working with obese patients is limited [25, 26]. It is essential to consider the presence of risk factors for complications [27]. The perioperative care of patients with body mass index (BMI) ≥ 30 is challenging and more multifaceted than for non-obese patients [28, 29]. However, to our knowledge, there is a lack of research focusing on the experiences of registered nurse anaesthetists in providing perioperative care to patients with obesity. Therefore, we have analysed how registered nurse anaesthetists positioned themselves toward obese patients, who are generally considered to be stigmatized in society while striving to provide equitable care.

In this study, the aim was to analyse how registered nurse anesthetists positioned themselves regarding obese patients in perioperative care.

## Methods

### Data collection

#### Participants and recruitment

The study setting was a university hospital in Sweden with operating clinics including orthopaedics, general surgery, gynaecology/obstetrics, neurosurgery, ear/nose/throat, paediatrics, and thoracic surgery. Registered nurse anaesthetists (RNAs) experienced in caring for obese patients in surgical procedures were invited by the first author to participate in this study. Information letters were sent out via e-mail to all RNAs at the clinics after approval by the Head of the Department. The interviewees were informed that participation was voluntary, that they could cease participation at any time, and that they would be anonymous and their names would not be traceable in the reports. They were also informed that audio-recorded interviews would be handled with confidentiality and that no one outside the research group would have access to the coded audio material. Fifteen RNAs, comprising nine men and six women aged between 31 and 63, participated in the focus group interviews. They had between 2 and 38 years of working experience as an RNA, with the majority having worked for more than five years.

#### Focus-groups interviews

Focus group interviews were conducted by MH and two research assistants in October 2021. The interviews took place near the participants’ work during working hours. The participants were divided into four focus groups, consisting of 3–4 participants in each group. The interviews were semi-constructed and started with a short introduction. After the introduction, the interviewers introduced a vignette that encouraged the respondents to reflect upon their experience of the perioperative care of obese patients; *How do you plan and prepare before meeting the patient in the operating theatre?* and *What are your thoughts when you meet the patient?* (Supplementary file 1). Vignettes are short descriptions of persons, situations, or events that are relevant to a study; they present hypothetical scenarios to allow discussion of issues that may be sensitive, ethically problematic, or challenging for the interviewees to interact with [30]. In this study, the vignette was designed as a case based on authentic patient data, including previous illnesses, height, weight, ASA classification, post-operative care needs, type of anaesthesia, and intraoperative monitoring (blood pressure, electrocardiogram, saturation). The case was tested and discussed with a small group of RNAs who did not participate in the study (Supplementary file 2). Focus group interviews were considered suitable since they are a way of approaching social and cultural constructions that can be changing and partly multifaceted, as well as people’s contradictory ways of understanding and relating to their lifeworld [31]. Focus group interviews make it possible to conduct an in-depth discussion about a particular area of interest and on the meaning-making or ‘the common’ construction of meaning, which takes place within the group [31]. The focus group interviews were moderated by the first authors while one of the research assistants took field notes. When needed, the interviewers asked follow-up questions for clarification. Each interview lasted approximately 30–40 minutes.

### Data analysis

The interviews were recorded and transcribed verbatim. Initially, the authors (MH, LL, and MJ) read through the transcriptions individually in search of patterns in the empirical material based on subject positions and interpretative repertoires [32–34]. An interpretive repertoire can be described as a recognizable way of describing or talking about a phenomenon and how the speaker positions themself verbally to others [33]. The analysing process began with a close reading of transcribed interviews and the coding was initially inductive and descriptive. After that, occurring themes or ways of talking were identified. Keywords and recurring themes were grouped together with an interpretive approach to gain into what was being said and *how* it was said [35]. Repertoires can be flexible and contradictory. Edley (2001) uses “subject position” as a central concept that defines the individual’s “location within a conversation” [36]. The individual positions become relevant within a specific conversation and make it possible to negotiate multiple subject positions [32]; that can vary both within a conversation and between conversations. This means that individuals position themselves or others in a preferable position (untroubled) or a non-preferable position (troubled) [32, 34, 37]. Throughout the analytical process, the authors regularly discussed the analysis and results. The research process was abductive, combining induction and deduction moving between the empirical material and [37]. Subsequently, interpretive repertoires were identified by, in more detail studying discursive constructions in relation to subject positions [30] and ideological dilemmas [38]. In society, ideology can be described as everyday “common sense” where repertoires can be contradictory and ideological dilemmas occur [38]. Billig et al. (1988), describe ideological dilemmas as being embedded in different forms of knowledge. Scientific knowledge and scientifically trained expertise have high value and are guarantors for facts and evidence in medical contexts, alongside experience-based knowledge gained from long clinical experience. This can produce dilemmas between competing types of knowledge. According to Billig et al. (1988), people ideologically share the same social and cultural beliefs based on the history that produced them.

## Results

Our analysis revealed that the RNAs were flexible in their talk about obese patients and positioned the patients in two troubled positions: the *untypical patient* and *the resource-demanding patient*. On the one hand, they positioned these patients in an untroubled position and expressed that the patients underwent the same perioperative procedures and received the same anaesthetic drugs and equipment, regardless of weight. At the same time, the RNAs positioned obese patients as troubled, and talked about them as untypical and the care as more challenging.

### The untypical patient

The interviewees talked about obese patients as untypical in relation to “normal” patients.

You don’t want to let them [the obese patients] understand that there is something different [about the way we handle the patients], so they feel we’re doing things differently with them. (FG 3)

In the excerpt above, the RNA expressed that they wanted obese patients to feel that they were being treated equally, indicating that even if they themself experienced specific difficulties, this should not affect the patients’ experiences.

In the excerpt below, the interviewees continued to talk about general societal values that positioned obese patients as troubled.

IP 1: Because I think this is such a thing in society that’s a bit taboo that patients might think, “I shouldn’t weigh this much.”

IP 2: Though maybe they shouldn’t. (FG 2)

IP 1 expressed that obese patients had internalized societal values about being overweight; that is, that they “shouldn’t weigh this much”. It is interesting to note that IP 2 responded with a rhetorical statement — “maybe they shouldn’t” [weigh this much] — indicating a belief that obese individuals are responsible for their obesity. Societal values can also affect personnel working in healthcare. The interviewees continued to reason about obese patients, comparing them to individuals with addictions.

Yes, to ask a person with an addiction to take responsible, for example, how many opioids they take. A person who’s used to lying about that sort of thing, I can ask that question and say that the reason I’m asking isn’t to moralize or something, but while you’re having surgery, I’m going to give you opioids. I need to know how much you need, because you’re tolerant, and I want to know how tolerant [you are], so I can give you the right dose. I’m not here to make you sober, but we’re here to make you feel safe during the surgery. It usually works without a problem. Then even old addicts usually do [tell you]; then you get it right, you get good answers. Not to offend obese patients, but they’re “kindred spirits”. But I think it happens easily. (FG 2)

In the excerpt above, the RNA described how patients with drug addiction were perceived as lying about their addiction. During surgery, patients receive opioids, and there can be complications in giving the correct dosage of opioids if the anaesthesia personnel are not aware of the patient’s addiction. The RNA compared the behaviour of patients with addictions to that of obese patients, using the phrase “kindred spirits”.

Yes, so I think like this with smokers and being overweight and like maybe high alcohol consumption, it’s all these groups, like, if it was easy to quit or to not be like that, they would have done it a long time ago. That’s it, so it’s a tricky one. (FG 2)

Some of the interviewees believed that obese patients had addiction problems, and that they lied to themselves and others.

You’ve also experienced patients who estimate their weight at 80 [kilograms] even though it’s probably double that, which is a concern. (FG 3)

This statement can be interpreted as a perception of obese patients as unreliable. Before the surgery, the patient is asked about the weight. In those cases, the patient knows the weight it is not always checked. This could be interpreted as it may be sensitive to question the credibility of the patient. However, an inaccurate estimate of a patient’s weight can be a concern since the medication given during surgery is based on body weight.

The interviewees described how women both apologized for their weight more than men, and were more likely than men to express guilt and shame when they realized that their weight exposed the staff to heavy work.

No, I haven’t been involved in that. But, oh! I’ve seen that there are sometimes apologies like “Sorry I’m so fat,”… and mostly, or exclusively, it’s been women who have apologized for themselves, in my experience. (FG 2)

I don’t think so. In that case, it’s the women, no. I don’t believe that men are usually ashamed of themselves; that’s my opinion, when you meet them.

Yes, but I think that maybe it’s more women who tend to apologise for themselves that, yes, they don’t fit on the table or things like that, or that it’s heavy for us, maybe, or something. (FG 1)

But I don’t think I’ve ever heard a man say, “Oh, I’m sorry, this is going to be difficult for you,” no. (FG 1)

The excerpts above can be interpreted as meaning that the RNAs expected to receive apologies from obese patients, and that obese male patients were a more challenging group than obese women since they did not express shame and guilt in the same way.

Concerns about risks also involved the children of obese patients. The RNAs talked about the risk of growing up with obese parents, suggesting that this could harm the children’s health because children of obese parents are more likely to become obese themselves.

Now we’ve talked about adults, I think it’s deplorable when you see obese children, I think it’s on the verge of writing a report of concern because they probably are poorly cared for at home. (FG 2)

And then you can imagine what their [the children’s] dental status is. They’ve grown up on pure sugar. So I usually mention this to the dentists from time to time, that you should write a report of concern if they come in here several times. because I think you should do it for the sake of the child. (FG 2)

Overall, the interviewees positioned obese patients as being different from “normal” patients. They positioned these patients as troubled, since they believed that the patients had addiction problems, were lying, and were endangering their children’s health. Because of this, their statements can be interpreted as meaning that the RNAs expected the patients to apologize for being too heavy and demanding for the healthcare system.

### The resource-demanding patient

In the RNAs’ narratives, the “untypical patient” was described as more resource-demanding than “normal” patients.

When you know you’re going to meet such a big patient. But spontaneously, I mean, if you have these measurements [size and weight] — in terms of resources, they require a lot; it’s not like a normal person. (FG 3)

The interviewees talked of the accurate preparation before anaesthesia and surgery was more complicated for obese patients than for “normal” patients. They explained that even though they carefully prepared the anaesthetic drugs and equipment before the patient arrived at the operating theatre, it was difficult to precisely predict the distribution of fat over the patient’s body; for example, whether it was more prominent around the waist, hip, or neck.

Sometimes it feels like you always have to come up with a [new] plan every time for patients who are severely obese. (FG 3)

In the excerpt above, the RNA described how the meeting with the patient might require extra time if the plan had to be changed. The need to change and replace necessary equipment was described as time-consuming and as increasing the personnel’s workload, which carried a risk of delaying the surgery. However, changing the equipment was also described as a common situation for the RNAs, as one of them explained: “But we’re used to changing the equipment, and we also do it for those [patients] who are shorter than we expected.”

When at the operating theatre, the RNAs experienced that the patients sometimes underestimated their weight, or gave a very out-of-date number. Heavy lifting was required when personnel in the operating theatre needed to position the patients correctly on the operating table.

But I feel that there doesn’t have to be very much weight before it starts to be difficult for us. It [the overweight] becomes a risk pretty fast. There’s probably no limit [for the overweight] you can set for everyone…… so it [the weight] can tell 85 kg, so it’s not always what you expect. So you should probably be a little vigilant, [the patient] can be overweight even though they [the patient] don’t weigh that many kilos. Then you don’t know if you have an estimated weight or if you’ve actually weighed the patient. (FG 3)

The obese patient was indirectly positioned as a burden in relation to those whom the RNAs described simply as “patients”. Exposure to heavy lifting increased the risk of jeopardizing the professionals’ health, due to the considerable risk of being injured during heavy lifts.

I also think we don’t have real devices for cases like this. I’ve hurt myself several times on these patients, and been on sick leave for probably six months during my working life after having broken [my back] myself on fat patients. (FG 2)

In the excerpt above, the RNA talked about the lack of devices to facilitate heavy lifting in the work environment. As the RNA described, the personnel’s work position for lifting heavy patients could be constrained and might cause work-related injuries.

IP: No. And you don’t get compensation from the county council for that, so it’s unfortunate.

I: But has it improved if you compare 20 years ago?

IP: No, I don’t think so. (FG 2)

Such injuries were not classified as work-related injuries. In the interviews, the RNAs described not having enough support from their managers when they needed extra resources such as “helping hands” from colleagues. They perceived a feeling of limited possibilities to do their best, and felt frustration on behalf of their obese patients in the organization.

I: How is it, is everything taking longer? Is it something that you expect, or….

IP: I don’t know what to say. It’s not like the surgery program is adapted to you because you have a severely overweight person, I’ve never experienced that. (FG 3)

In the focus groups, the interviewees also included a socioeconomic perspective. They expressed ambivalence over whether they should ask the patient if they knew they were jeopardizing their health by being obese since they were afraid that the patient would feel singled out.

It doesn’t feel that obvious to give such information about the risks of being overweight, I wouldn’t dare to say that. But it might also have to do with the fact that you don’t want to offend [the patient]. So, you maybe, it’s a little hard to say it straight to a patient as well. I don’t feel comfortable asking, “Have you heard about the risks of being overweight?” I don’t dare to ask the patient. (FG 3)

[…] obviously much more expensive than a normal person is, if you can say so […] It’s both equipment, people, and money. I mean, you have twice as many personnel. Of course it’s more expensive. (FG 3)

In the excerpt above, the RNA not only pointed to the costs of the surgery but also included the cost of extra personnel and equipment.

Overall, the interviewees experienced the care of obese patients as being more time-consuming and demanding than care for the “normal” patient. More resources such as time, personnel, and special equipment were needed. The patients were positioned as troubled, since the care was expressed as being more harmful to the staff due to heavy lifting.

### Dilemmas in communication

The care of obese patients was described as heavy and more physically demanding not only for the RNA but also for all personnel involved at the operating theatre. Feelings of frustration elicited emotional reactions in the RNAs which they did not want the patient to perceive. In the excerpt below, the interviewee described sighing, not in front of the patient but before meeting the patient.

IP: Sometimes I think that there can be a little sighing, that’s like a heavy patient, and it’s physically heavy. And then I feel sorry for the patient because often when you enter a [operating] room, and it isn’t prepared, then you realize that the planning has been terrible, and “oh we need this, and we need that,” and the patient is constantly reminded that this is because I’m too big, this is because I have a high BMI. And it takes time, they can’t find the equipment, and it’ll be like that, yes, it’ll be a bit uncomfortable socially, so it’ll be like that.

I: Even if you don’t explicitly say [to the patient] that it’s their fault, but that….

IP: Yes, it feels like the patient, and this is my prejudice, but it feels like the patient thinks that it’s because they have a high BMI. (FG 2)

Weight was perceived as a sensitive subject in perioperative care. The RNAs said that most patients did not mention their weight. However, they felt that the atmosphere in the operating theatre was less tense when the patients openly talked about their weight, with straightforward communication.

Then there are some [patients] who say that they are overweight as if somehow they have no problem with it and have accepted it more or less. I try to think a little about what I say, but sometimes it releases [the tension] a bit if they [patients] aren’t so sensitive about it. (FG 3)

Another way to handle the sensitive interaction was to use humour to de-dramatize the situation for the patients. Humour has been described as a tool for reducing distress, anxiety, stress, and tension in stressful situations.One interviewee narrated how humour could de-dramatize a tense situation.

I think that, as an obese person, I have a particular advantage when it comes to treatment. In part, I can, with a twinkle in my eye; yes, like, yes, we’re in the same boat. This kind of extra preparation, and if there’s going to be support, additional work, and stuff that it’s quite obvious even to the patient is about them being big, so I say yes, but it’s because you’re our size, then you get VIP treatment, you’re supposed to be safe with this. We invest, we don’t scrimp on anything, but it’s… trying in some way to turn the preparatory work into something positive. Because, I mean, the patient is fully aware that they are obese, I’m fully aware that I am obese. That’s the situation, and you don’t need… I don’t moralize about it, but, I don’t know… somehow try to keep the mood light. So I don’t know that it’s taboo; for my part, yes, I don’t experience it that way. (FG 2)

Overall, the interviewees talked about the difficulties of talking straightforwardly with the patients about their weight. However, in situations when the patients talked openly about their weight, they did not position themselves as troubled and the RNAs experienced the atmosphere as less tense. Another way of de-dramatizing a tense situation was to use humour that opened a way to communicate with the patient. Goffman (2009) means that humour can be used as a coping strategy, and joking can save the social situation and it might lighten the mood for the moment.

## Discussion

In this study, we explored how RNAs constructed dilemma-repertoires of obese patients in perioperative care. Treating all patients equally was said to be important, and the RNAs stated that obese patients should receive the same perioperative care as “normal” patients. However, the RNAs described obese patients as “untypical” and the care of this patient group as more “resource-demanding” than care for “normal” patients. This created conflicting feelings toward the patients, generating frustration over the extra work. Situations which involve competing and conflicting types of knowledge are described by Billig et al. (1988) as “ideological dilemmas”. For the interviewees in this study, an ideological dilemma arose as the RNAs strived to treat patients with equity, but at the same time described obese patients as untypical and resource-demanding; they thus experienced conflicting feelings towards these patients. Robstad et al. (2018) found that intensive care nurses expressed frustration relating to the physically demanding care situations and an unwillingness to care for such patients among some colleagues [39]. An earlier study of professionals in primary care found that these professionals considered equity as an “extra” element in care that pushed the margins of their day-to-day practice, and the authors concluded that it is essential to make visible the competing discourses that interact within the organization; that is, within healthcare [40].

However, the RNAs also described frustration stemming from their belief that obesity is self-inflicted. Billig (1988) argues that “common sense” is reflected in society and organizations, meaning that the members of a society reflect that society’s “common sense” in everyday life at their workplaces. Alberga et al. (2016) concluded that healthcare professionals need to be aware of their attitudes and behaviors toward obese patients, and how these negative stereotypes can affect patient care and staff commitment [41]. In order to overcome the weight stigma that permeates society, it is necessary to change the overall structures and social norms that support and maintain this stigma [41]. Biased attitudes regarding weight have been identified among university students in health science disciplines [15], and so it is essential to ensure that future healthcare professionals develop greater awareness and understanding of the potential influence of weight bias and how negative attitudes influence the patient-provider relationship.

The interviews included narratives of patients who apologized and were embarrassed about their size; all of these patients were women. To de-dramatize the care situations, the RNAs talked about “acting professionally” to make the patients comfortable. One specific coping strategy that was described for lightening the mood was to have a humorous attitude toward the body of the obese patient. As Goffman explains, if the stigmatized person notices that others have difficulty ignoring the stigma (in this case, obesity), it can be positive to joke about it and thus save the social situation [21]. Still, although joking about one’s weight might lighten the mood for the moment, in the long run it can reinforce the feeling of stigma [42]. Humour can be used as a coping strategy, and self-distancing or self-acceptance can ease the situation for those around the individual. The use of humour can be a way for those in lower status positions to express resistance to the more powerful [43, 44]. Mazurkiewicz et al. found that severely obese women used self-deprecating humour to reduce stress, especially when they felt stigmatized [45].

When RNAs face ideological dilemmas between their beliefs about larger-bodied individuals and the goal of treating all patients equally, they risk consciously and/or unconsciously blaming the patients via verbal and nonverbal communication. Caring for patients with specific characteristics may need extra time for preparation, and the RNA may have to work faster to avoid delaying the planned schedule at the operating theatre. In the realm of healthcare, efficiency has significantly influenced the discourse among healthcare providers, highlighting the importance of time pressure and care processes. However, this emphasis on efficiency can sometimes lead to compromises in best practices, fostering a tendency towards a production-line mentality within the healthcare system [46, 47]. Frustration can arise when RNAs experience a lack of support from their managers to deliver equal care for all patients.

In this study, we have explored how RNAs constructed repertoires in perioperative care. Treating all patients equally was considered vital, and the RNAs stated that obese patients should receive the same perioperative care as “normal” patients. However, they also described obese patients as “untypical”, and perceived the care of these patients to be more “resource-demanding” than care for “normal” patients. This created conflicting feelings toward the patients, and generated frustration over having to perform extra work. According to the Swedish healthcare system, personnel are obliged to provide equal care and respect for all individuals. Nevertheless, healthcare must be organized so that it promotes cost-effectiveness; this can be perceived as contradictory as our findings demonstrate.

## Conclusions

In this study, we explored how RNAs constructed repertoires of obese patients in perioperative care. Treating all patients equally was considered vital, and the RNAs stated that obese patients should receive the same perioperative care as “normal” patients. However, they also described obese patients as “untypical”, and perceived the care of these patients to be more “resource-demanding” than care for “normal” patients. This created conflicting feelings toward the patients, and generated frustration over having to perform extra work. According to the Swedish healthcare system, professionals are obliged to provide equal care and respect for all individuals. However, healthcare must be organized so that it promotes cost-effectiveness; this can be perceived as contradictory, as our findings demonstrate.

### Methodological considerations

In this study, we used focus group interviews to elicit descriptions and narratives that were discussed and reflected on in groups [48]. The use of focus group interviews makes it possible to achieve an in-depth discussion about a particular area of interest and the meaning-making or “common construction of meaning” that takes place within a group. People have contradictory ways of understanding and relating to their way of living. Focus group interviews are thus a way of approaching both social and cultural constructions which are partly multifaceted.

To confirm the trustworthiness of the findings there are some concepts to discuss. To increase the *credibility* and *authenticity* of the analysis, excerpts were inserted into the text to verify the accuracy of the findings. In addition, the participants varied in age, gender, and current employment experience. The findings in this study should be *transferable* and consistent and could be replicated (*dependability)* to operating clinics in other countries since obesity is a growing health problem globally and RNAs face these patients in daily work.

### Research limitations

We interviewed RNAs from Sweden, a country in northern Europe with a public healthcare system. However, we believe that the results of this study can be considered a global contribution to a discussion of the importance of equity in healthcare among staff and students in healthcare professions.

In this study, the interprofessional research team has experience in both nursing and social work MH (RNA) and LL (Critical Care Nurse) have a combined position with the operating clinic, MJ is a sociologist and has no connection to health care. This was an important aspect throughout the analytical process. One of the authors (MH) has previously been a colleague to some of the informants. During study planning, the potential effects of interviewing within one’s organization and competence area were discussed [49]. Hence, the involvement of MJ, a sociologist with no connection to health care, was proved advantageous in promoting confirmability of the study. This was an important aspect throughout the analytical process. In the process of analysing the interviews, the interpretation of the texts was repeatedly discussed with the research team. Understanding the medical terminology was also an advantage when transcribing the communication and during the process of analysing the text and the observations. Wetherell & Potter (1992) argue ”At its most basic, an analyst has to have a basic comprehension of what the words in a language mean to make sense of a text or transcript” [50].

### Electronic supplementary material

Below is the link to the electronic supplementary material.

**Supplementary file 1**: Interview questions

**Supplementary file 2**: The Vignette

## Data Availability

The interview data analysed during the current study are available from the corresponding author on reasonable request.

## Abbreviations

RNA: registered nurse anaesthetist
HIV: human immunodeficiency virus
