# Supplementary material for: Can equity in care be achieved for stigmatized patients? Discourses of ideological dilemmas in perioperative care
Source: BMC Health Serv Res. 2024 Feb 15;24:210. doi: 10.1186/s12913-024-10580-5 (PMC10870466; doi:10.1186/s12913-024-10580-5)
Supplement: Supplementary file 1 — Interview questions [file 12913_2024_10580_MOESM1_ESM.docx]

**Supplementary file 1. Interview questions**

Previous research has indicated that individuals who are overweight or obese may encounter healthcare treatment that is perceived as hurtful, offensive, or discriminatory. Instances have been described where medical equipment and the physical environment are not adequately adapted for obese patients. Conversely, nurses have highlighted the challenge of establishing mutual trust in relationships with obese patients due to the stigma associated with their weight. Nurses express concerns about inadvertently expressing themselves inappropriately or insultingly towards overweight individuals.

When You are responsible for this patient (below) in the operating theatre:

- *How do you plan and prepare before meeting the patient in the operating theatre?*
- *What are your thoughts when you meet the patient?*
