# Supplementary material for: Can equity in care be achieved for stigmatized patients? Discourses of ideological dilemmas in perioperative care
Source: BMC Health Serv Res. 2024 Feb 15;24:210. doi: 10.1186/s12913-024-10580-5 (PMC10870466; doi:10.1186/s12913-024-10580-5)
Supplement: Supplementary file 2 — The Vignette [file 12913_2024_10580_MOESM2_ESM.docx]

**Supplementary file 2.** **The Vignette**

**Case**

The patient is scheduled for a hysterectomy, possibly laparoscopic, with ureteral placement.

**Medical History**

The patient is a severely overweight woman with hypertension and type 1 diabetes. Otherwise, she is in good health.

- Length: 167 cm
- Weight: 146 kg.

Dosing weight: 70 kg.

Preoperative assessments: Echocardiography and spirometry have been conducted.

**Anesthesia summary**

The American Society of Anesthesiologists (ASA) classification: 3-3: (Patient with serious illness)

Medical resource: 4

Preferred form of anesthesia:

- General
- Spinal anesthesia (using an extra-long spinal needle)

Pre-anesthesia preparations:

- Arterial needle (blood gas checked on room air)
- Ramping (positioning with a special cushion)
- Pre-oxygen before intubation with a video laryngoscope
- Oxygen with peep and pressure support
- Central venous catheter

Monitoring during anesthesia:

- Hourly diuresis check
- Electrocardiogram
- Saturation monitoring
- Arterial pressure monitoring
- Central Venous Pressure monitoring
- Temperature measurement

Accesses:

- Peripheral Venous catheter
- Arterial catheter
- Central venous catheter
- Nasogastric tube
- Urinary catheter

Postoperative Care requirements: Overnight stay anticipated. Check with the ICU before administering anesthesia.
